# Building better yeast

**DOI:** 10.1038/s41467-018-04159-y

**Published:** 2018-05-22

## Abstract

The Sc2.0 project has set out to synthesise the *Saccharomyces cerevisiae* genome, with each chromosome redesigned along agreed principles. In this collection of papers, the researchers involved show how SCRaMbLE—Synthetic Chromosome Rearrangement and Modification by LoxP-mediated Evolution—can be used to rapidly reorganise the genome.

The genome sequence and what can be learned from its wealth of information occupies the core of modern biology in much the same way a physical genome lies at the heart of a cell. The sequence of DNA organised into genes, arranged along the chromosomes together with repetitive elements, structural features and remnants from history are acted on and shaped by evolution which sorts through variation and mutation to select against organisms that cannot adapt to their environment. The past 50 years of molecular biology has given us the tools and understanding to manipulate genomes, ushering in an age of synthetic biology, where genetic variation can be designed and evolution harnessed and accelerated.

“By exploring the huge amount of genetic variation generated, in just a few days researchers can generate new strains for laboratory or industrial applications”

In the spirit of Richard Feynman’s words, “what I cannot create, I do not understand,” an international team of scientists has been working to synthesise a *Saccharomyces cerevisiae* genome as part of the Sc2.0 project. As part of the project, the synthetic chromosomes would have minimal alteration to the sequence of the genes themselves but the architecture of the genome would be engineered along shared design principles. To date, six of *S. cerevisiae*’s sixteen chromosomes have been redesigned to remove transposable and repetitive elements, the subtelomere regions and—in a radical approach that takes this endeavour beyond merely tidying up a naturally messy genome—moving the tRNA genes to a de novo chromosome and inserting loxPsym recombination sites in between genes and chromosomal structural features^1^.

It is these loxP sites, which facilitate the recombination of genetic elements in either direction by the Cre recombinase, that allow the synthetic chromosomes to be used for applications that require large amounts of genetic diversity. The method, SCRaMbLE—Synthetic Chromosome Rearrangement and Modification by LoxP-mediated Evolution—is initiated by the induction of Cre expression. The synthetic chromosomes undergo large-scale re-arrangements leading to deletions, duplications, inversions and translocations. SCRaMbLE, published in *Nature* in 2011, was first demonstrated in partially synthetic chromosomes synIXR and semi-synVIL^2^. The strains displayed large variations in growth rate and later analysis using deep sequencing on the SCRaMbLE strains showed they contained complex rearrangements that were limited to the synthetic chromosomes^3^.

To facilitate discovery of current and future advances and applications of the Sc2.0 project we have launched a collection of papers published by *Nature Communications*. In this first batch of papers, the researchers in the Sc2.0 project show the power of SCRaMbLE to generate yeast strains for product production and to refine the system with an expanded set of tools to control SCRaMbLE and investigate the strains generated. By providing selective pressure following the rearrangement of the genome, the groups can use SCRaMbLE to explore the diversity of genetic space, identifying strains that can survive hostile conditions or produce high titres of desired product.

For example, when applied to yeast with a synthetic chromosome V SCRaMbLE has been used to generate strains with improved violacein or penicillin biosynthesis, or improved xylose utilisation, demonstrating that SCRaMbLE is applicable to the improvement of diverse and unrelated pathways^4^.

The principles of SCRaMbLE have also been transferred from the cell to the test-tube—the in vitro SCRaMbLE^5,6^. By taking a pathway or set of genes away from the complexity of the genome, SCRaMbLE can be used to rapidly prototype variations in a single pathway.

However the power of SCRaMbLE to generate a large amount of genetic diversity can be a double-edged sword, as potentially highly desirable recombination events can be selected against if they impose a severe growth defect. Mating yeast bearing synthetic chromosomes with wild-type *S. cerevisiae* or closely related species to generate diploid strains^7,8^ compensates for these ill effects leading to more robust strains and expanding the application of SCRaMbLE to industrially relevant yeast strains.

The original SCRaMbLE method can be extended to increase strain diversity and aid in their identification. For example, MuSIC—Multiplexed SCRaMbLE Iterative Cycling—sends yeast through five rounds of SCRaMbLE to drive increases in catenoid titres^7^. ReSCuES—Reporter of SCRaMbLEd Cells using Efficient Selection—rapidly screens for successfully SCRaMbLE affected yeast by integrating two selective markers, one of which is functional before SCRaMbLE and one only after the Cre recombinase inverts their orientation^9^. To improve recombination control, light has been harnessed to control Cre, in what is referred to as L-SCRaMbLE^10^.

SCRaMbLE is a powerful example of the many potential innovations made possible by rationally designing a well understood species at the fundamental level of the genome. By exploring the huge amount of genetic variation generated, in just a few days researchers can generate new strains for laboratory or industrial applications. As Sc2.0 nears completion, the number of synthetic chromosomes available will increase, further expanding the genetic landscape that can be accessed and reshuffled. In addition to the industrial applications, Sc2.0 and SCRaMbLE can be focused back towards basic research to aid researchers to understand what genomic features and genetic combinations are—and importantly are not—necessary for a viable cell. The principles behind SCRaMbLE, including the in vitro prototyping of rearranged pathways, could be expanded to other industrially important species. With this in mind, SCRaMbLE joins other techniques for genetic manipulation as a powerful tool that harnesses the potential diversity found in the genome.
